# The role of professional networks and institutional prestige in shaping the first career moves of scholars

**DOI:** 10.1093/pnasnexus/pgag168

**Published:** 2026-05-15

**Authors:** Alexandra Rottenkolber, Ola Ali, Gergely Mónus, Jiaxuan Li, Jisu Kim, Daniela Perrotta, Aliakbar Akbaritabar

**Affiliations:** Institute for Analytical Sociology, Linköping University, 60174 Norrköping, Sweden; Department of Digital and Computational Demography, Max Planck Institute for Demographic Research, 18057 Rostock, Germany; Department of Digital and Computational Demography, Max Planck Institute for Demographic Research, 18057 Rostock, Germany; Complexity Science Hub, 1030 Vienna, Austria; Department of Digital and Computational Demography, Max Planck Institute for Demographic Research, 18057 Rostock, Germany; ANETI Lab, Corvinus University Budapest, 1093 Budapest, Hungary; Department of Digital and Computational Demography, Max Planck Institute for Demographic Research, 18057 Rostock, Germany; Department of Sociology, University of Oxford, Oxford OX1 1JD, United Kingdom; Department of Digital and Computational Demography, Max Planck Institute for Demographic Research, 18057 Rostock, Germany; Department of Interdisciplinary Social Science, Utrecht University, 3584 CH Utrecht, The Netherlands; Department of Digital and Computational Demography, Max Planck Institute for Demographic Research, 18057 Rostock, Germany; Department of Digital and Computational Demography, Max Planck Institute for Demographic Research, 18057 Rostock, Germany; Institute of Sociology and Demography, University of Rostock, 18057 Rostock, Germany

**Keywords:** scientific mobility, science of science, co-authorship networks, bibliometric data

## Abstract

Mobility of researchers is closely linked to knowledge diffusion, scientific innovation, and international collaboration. While prior research highlights the role of networks in shaping migration flows, the extent to which personal and institutional ties influence the direction of scientific mobility remains unclear. This study leverages large-scale digital trace data from Scopus, capturing the complete mobility trajectories, co-authorship networks, and collaboration histories of 172,000 authors over two decades (1996–2020). Using multinomial and conditional multinomial logit models, we examine scholars’ first career move by (i) classifying moves into four network-defined mobility-type categories and (ii) modeling destination choice as a function of co-authorship connection strength, institutional linkages, and institutional prestige. Our findings show that not only first- but also second-order co-authorship ties—connections to a scholar’s collaborators’ collaborators—are a strong correlate of the direction of a move. Scholars with extensive individual professional networks, particularly those migrating abroad, are more likely to move along individual ties. In contrast, scholars from prestigious institutions, and those moving within national borders, are more likely to follow institutional routes. The destination-choice models confirm that both individual and institutional ties are associated with a higher probability of moving to specific research institutions, with a larger estimated association for individual than for institutional ones. Overall, this research provides empirical evidence on how individual and institutional connections shape scholars’ first career mobility. The findings have important implications for migration theory and policy, emphasizing the need to support both individual and institutional collaboration networks to foster global scientific and knowledge exchange.

Significance Statement Understanding scholars’ mobility between institutions is important for understanding scientific innovation and the global research system. Although both professional networks, individual and institutional ones, and prestige shape academic mobility, their roles have often been conflated. This study disentangles these relationships, revealing that prestigious institutions attract talent with less dependence on prior individual professional ties, whereas less-prestigious institutions depend more heavily on such connections when recruiting scholars. We further show that individual connections are especially important for cross-border mobility, whereas national mobility is more often observed along institutional pathways. Together, these findings provide a clearer picture of how first academic moves unfold and how policies can better support global knowledge flows.

## Introduction

Scholarly migration is a significant and growing form of mobility that not only influences individual careers (1–3) but also shapes the development of entire research fields, institutional collaborations, and innovation systems (4–6). Scholars are key actors in the global flow of knowledge, and their movements across borders can accelerate the spread of ideas (7, 8), foster innovation (9), and strengthen international partnerships. The first mobility event in a scholar’s career, often around the transition out of the PhD, marks the entry into the academic labor market and is frequently realized through a first postdoctoral or faculty appointment. This transition can have lasting consequences for subsequent career trajectories (10). Understanding how co-authorship and institutional connections are associated with these moves is critical for informing policies aimed at enhancing scientific cooperation and knowledge transfer within and between countries.

Unlike many other migrant groups, scholars are extensively documented through publicly available data on their publications, collaborations, and career trajectories, which are increasingly used for demographic research at subnational and international levels (11, 12). Traditional migration data, such as census records, visa applications, and employment statistics or surveys, often lack the granularity needed to track scholars’ movements across institutions and countries. In addition, these data sources do not provide evidence on scientific collaboration networks that might influence mobility decisions. In contrast, digital trace data from bibliometric sources provides real-time, longitudinal insights into individual career trajectories, co-authorship networks, and institutional affiliations (13, 14), offering a level of detail and transparency typically not available for other migrant groups. By analyzing this rich data, we can better understand how academic networks and institutional partnerships shape the first career move of scholars, thereby contributing to broader theories of high-skilled labor mobility and the knowledge economy.

These same networks also offer a rare opportunity to empirically examine a longstanding insight from migration theory: that networks shape the opportunity structures individuals face when making mobility decisions (15). Both kin ties, such as family and friendships, and weaker connections to broader communities, help individuals navigate the migration process. These networks serve as channels for information and sources of social and economic support (16). Despite their importance, these network effects remain difficult to quantify (17). Sociological and anthropological studies of migration provide strong ethnographic evidence that social and institutional networks shape migration flows, often using diaspora communities to demonstrate how household-, community-, and macroeconomic-level contextual factors contribute to stable migration pathways between specific origins and destinations (18, 19). With regard to scholars, both co-authorship connections and institutional relationships (eg between universities or research organizations) play critical roles in facilitating movement (20). The availability of these networks, both individual and institutional ones, can shape career opportunities, access to research resources, and long-term professional success (21–27). This makes scholarly migration not only a relevant and increasingly significant phenomenon but also a unique case for analyzing migration through the lens of network effects. Which leads us to our first two research questions

RQ1: What role do individual-level connections play in shaping the first career moves of scholars? Specifically, do scholars preferentially move to institutions where they have prior co-authors or are connected to via their co-authors?
RQ2: Do institutional partnerships and connections influence the likelihood of scholars moving between specific institutions? Specifically, do scholars preferentially move to institutions that are connected to their home institution?

Under the assumption that both individual and institutional ties may affect mobility, the strength of these ties is likely to matter, too. Recent research has emphasized not only direct ties but also higher-order connections as influential in career advancement and information diffusion (28–30). However, it is unclear whether individual or institutional ties more strongly shape mobility decisions, and whether these effects are additive, nonlinear, or whether simply having “at least one” connection is sufficient. This leads to our third question:

RQ3: Do the effects of individual- and institutional-level connections on mobility outcomes scale with the strength of those connections?

Finally, the literature on academic stratification highlights the role of institutional prestige in structuring career trajectories (31–34). Prestige can influence who gets hired where and may condition how scholars are able to leverage their networks. Because high-status departments already attract a large applicant pool, they can screen candidates based on publicly visible merit signals, thereby reducing their dependence on personal referrals (35). For lower-ranked universities, the situation often looks different, and hiring through trusted co-author links can mitigate informational uncertainty (36). Following these lines of thought, we hypothesize that prestige moderates the use of networks. Likewise, scholars with larger personal networks may be better positioned to access desirable opportunities, while those with smaller networks may face structural constraints (12, 37). Together, these factors may structure scientific mobility. Thus, we ask:

RQ4: Do institutional prestige and the size of a scholar’s professional network moderate the relationship of a scholar’s embedding on the direction of first academic mobility?

To answer these research questions, we use a 2021 extract of Scopus data, which includes complete historical records of authors’ publications and affiliations up to the end of 2020. Mobility events are identified by analyzing changes in author affiliations at both subnational and international levels (12). We focus on the first career moves of scholars and the direction of mobility, which are key transitions that often shape academic trajectories in the long term (10, 38). Mid- or late-career moves are excluded here, as they could be influenced by family circumstances, life events, and gendered macro structural factors (39). We deliberately set aside both these moves and the equally important question of whether a scholar becomes mobile, which may be governed by different mechanisms and could be investigated in the future work. Instead, we focus on the first career move that is the entry point to the national or global academic labor market, likely to coincide with the first postdoctoral or faculty employment.

To assess the role of individual- and institutional-level connections on the first career mobility outcomes, we construct co-authorship networks at both individual and institutional levels. At the individual level, we measure first- and second-order ties between scholars—that is, direct collaborators as well as the collaborators of collaborators—and track their affiliations. We define the individual level connection strength as the proportion of a scholar’s co-authorship network that is affiliated with a given institution, where the network is defined as the set of unique first- and second-order co-authors (each co-author counted once, regardless of the number of joint publications).

**Figure 1:**
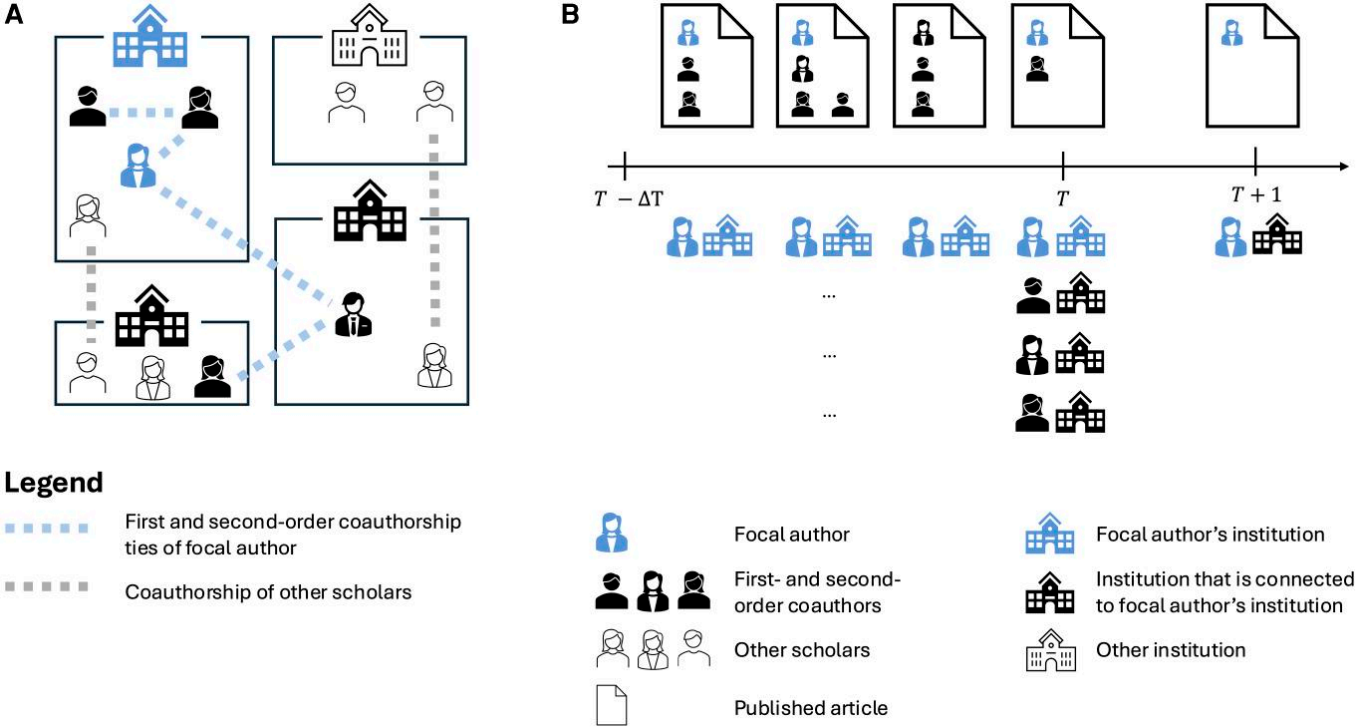
Illustration of the analytical strategy and retrospective measurement of scientific collaboration and mobility. A) Mapping individual-level and institutional-level collaboration ties. Visualization of the focal author’s individual connections to different institutions prior to the mobility event (blue dotted lines). We trace the focal author’s direct co-authors as well as the co-authors of co-authors, and record the institutions they are affiliated with. The institutional-level connections could be independent of the focal author and represented by the gray dotted lines, while those ties depending on the focal author are the blue dotted lines. The snapshot at time *T*, ie the year before the mobility event, is used to map past connections and calculate the respective connection strengths. B) Detecting migration events through affiliation addresses in publication records. Timeline showing the stream of papers published by both the focal author (in blue, on the top) and their first- and second-order co-author (in black, on the bottom) prior to the observed mobility event of the focal author at time T+1. All authors’ affiliations are documented for each publication, and a mobility event is recorded when the focal author’s affiliation changes consistently between different years.

At the institutional level, we assess collaborative links between institutions. The institutional strength measure captures the relative intensity of collaboration between two institutions, measured yearly by the number of joint publications scaled by the focal institution’s total collaboration volume across all partners. Figure 1 illustrates this analytical strategy. Our network variables capture co-authorship-documented professional ties, a conservative proxy for scholars’ broader social and mentoring networks (40, 41). Because many influential relationships, eg advisors, informal mentors, conference contacts, do not always culminate in joint publications, our estimates should be interpreted as lower-bound effects of network embeddedness (see Data and methods section for details). Institutional prestige is captured using the Leiden Ranking (42) (see Data and methods section for details).

To model mobility outcomes, we use logit models in two complementary specifications. First, we estimate multinomial logistic regressions that classify mobility events into four network-defined categories based on the presence of individual and institutional ties. Second, we model destination selection using a conditional multinomial logit (discrete-choice) specification that incorporates alternative-specific predictors, including continuous measures of individual and institutional level connection strengths. Both specifications control for institutional attributes (eg institutions’ prestige rankings, and geographic location). In the multinomial logit we additionally control for individual characteristics (such as gender, career age, and academic discipline). These types of individual-level covariates cannot meaningfully be estimated in the discrete-choice specification and are hence not part of the latter. Together, this design allows us to compare how individual and institutional embeddedness relate to scholars’ first career mobility.

## Results

All results are conditional on mobility: they describe how a researcher’s individual and institutional embedding is associated with the choice of destination, not with the prior decision to move at all. The decision to remain nonmobile could be studied in the future.

### Network size matters

**Figure 2:**
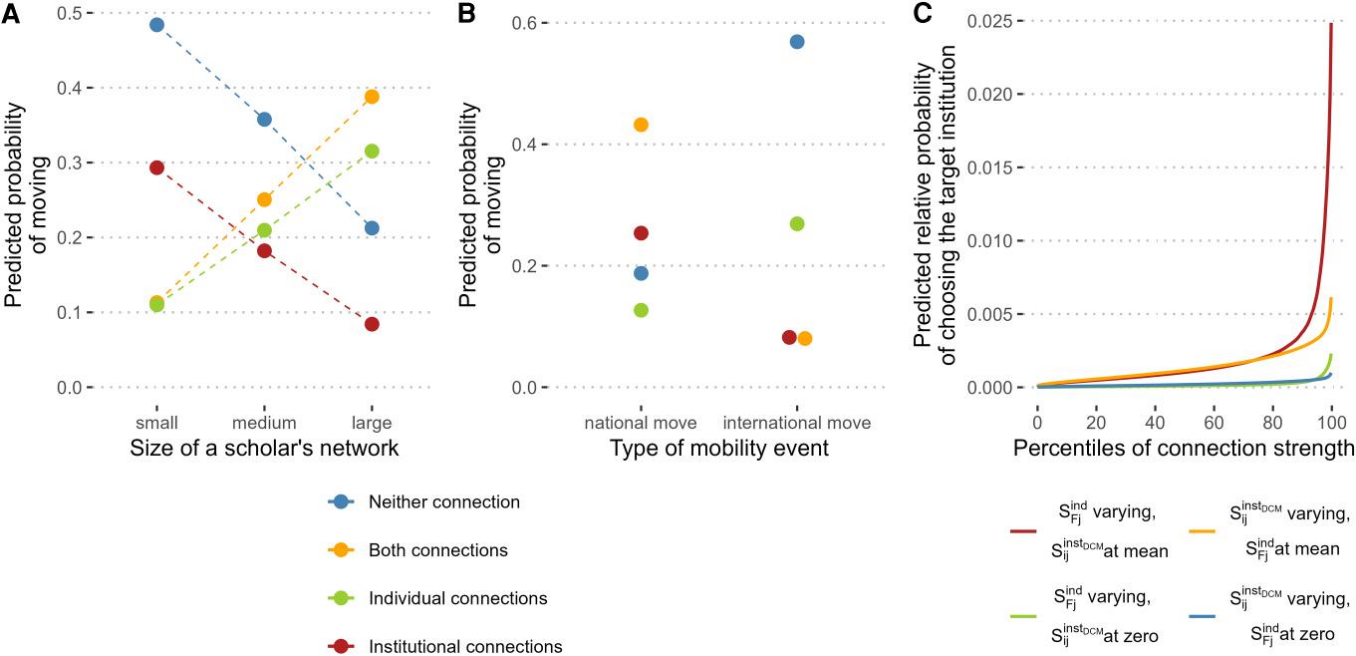
Effect of the size of a scholar’s network (A), the type of mobility event (B), and the strength of a scholar’s connection to a potential target institution (C) on the likelihood of a specific mobility outcome. A) Predicted probabilities of moving along one of the four pathways in the event of scholarly mobility plotted against the size of a scholar’s personal network (measured by first- and second-order co-authorship ties), as estimated by the multinomial logistic regression. While the likelihood of moving along individual ties or a combination of individual and institutional connections is associated with the size of a scholar’s network, the likelihood of moving along no ties or institutional connections decreases. B) Similar analysis as in (A), but here the predicted probabilities are plotted against the type of mobility, ie national or international. Whereas most scholars who move nationally do so along institutional ties or a combination of institutional and individual ties, it is most common for scholars who move internationally not to follow any connection at all, or if they do, they move along individual-level connections. C) Marginal relative predicted probabilities of choosing a certain alternative destination plotted against the percentile rank of the individual- and institutional-tie strength measure, all else equal, as estimated by a conditional multinomial logit model in the discrete-choice framework. The probabilities should only be interpreted in relative terms, not in absolute ones, as the absolute values are conditional on the number of options to choose from (ie the size of the choice set). The percentile ranks were only calculated for connection strength values that were different from zero, ie where a connection was present at all. The use of percentile ranks allows for the comparison of the individual and institutional strength measures. If a potential target institution is connected to an author via both individual and institutional ties, the probability of being chosen by the scholar is much higher than if only one of the two connection types exists. Furthermore, the probability of a potential target institution being chosen increases disproportionately at high percentile ranks, especially if both types of connections are present. Note that the plotted marginal predicted relative probabilities as shown in (C) should only be interpreted in relative terms. An interpretation in absolute terms is not meaningful here.

The size of a scholar’s individual professional network plays a crucial role in shaping the direction of mobility. Our multinomial models consistently indicate that the number of first and second-order connections—measured through co-authors and co-authors of co-authors—is among the most influential factors relating to scholars’ movement patterns. This finding is further supported by an additional analysis using a Decision Tree to predict mobility outcomes (see Supplementary material Part 2, Fig. S1 and Tables S2, S3, and S4). As shown in Fig. 2A, scholars with large personal networks are significantly more likely to follow individual connections or a combination of individual and institutional ties (see “individual connections” and “both connections” in the figure). In contrast, those with smaller networks mostly follow pathways unrelated to preexisting connections (ie “neither connection”). Scholars with medium-sized networks show a relatively higher likelihood of moving along institutional pathways compared to highly connected scholars and are more likely to rely on individual ties and a combination of both types of ties compared to those with smaller networks. Because the categories depend on prior ties, larger networks mechanically increase the likelihood that a tied destination exists; thus, the association with network size reflects both availability and selection.

### Strength effects

The destination-choice (conditional multinomial logit) models allow us to compare the relative salience of individual- and institutional-level ties in destination selection. The underlying choice sets, however, also include institutions with no prior connection. Figure 2C visualizes how the probability of selecting a destination institution varies with the strength of a connection. The effects are plotted conditional on a tie being present, since strength percentiles are defined only for nonzero connections. We observe a nonlinear relationship: When connected to an institution through only one type of tie (individual or institutional), a noticeable increase in the predicted probability of choosing that institution occurs only for connection strengths in the highest percentiles (80th percentile and above). In contrast, when both types of ties are present (ie a combination of individual and institutional ties, see red and orange lines), the likelihood of choosing an institution is associated almost linearly with connection strength up to the 75th strength percentile, and then rises more rapidly with especially strong ties. The strongest effect is observed when scholars have strong individual ties and an average institutional embedding (red line).

One note on Fig. 2C: the percentile ranks are computed only among nonzero ties, which visually accentuates the upper bound of our strength measure. In the data, connection strengths above 0.9 are extremely rare (0.4% of nonzero individual ties; 0.005% of nonzero institutional ties). Re-estimating the model after excluding these cases yields substantively unchanged results.

### Individual and institutional connections are differently relevant for national and international mobility

Mobility direction patterns differ significantly between national and international moves (see Fig. 2B). For national moves, the largest proportion of scholars follows a combination of individual and institutional ties, or, less frequently, institutional ties only, reinforcing the importance of overlapping personal and institutional ties. In contrast, international moves frequently fall into the “neither” category, with the highest predicted probability among all types (close to 0.6), indicating that many international moves occur outside established career networks. When ties do influence international moves, they are most likely individual rather than institutional ties, however, appear to play a limited role in shaping international mobility and are mainly at play in national mobility.

### Prestige conditions the role of connections

**Figure 3:**
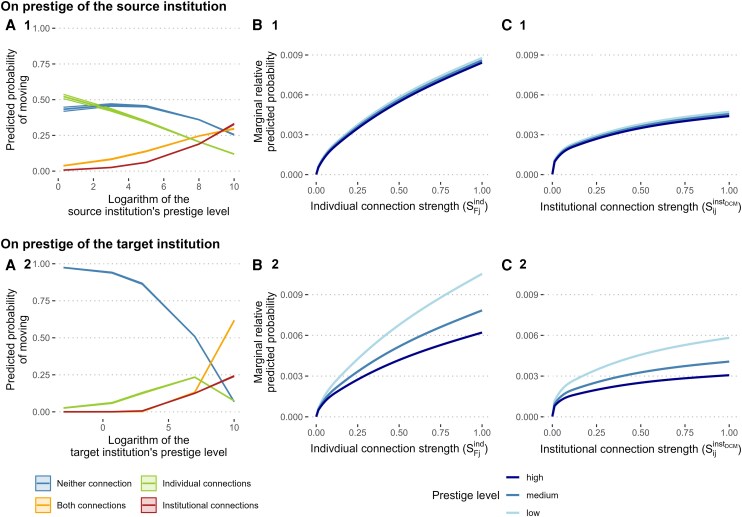
Effect of the prestige of an institution on the predicted probabilities to move from there or be chosen as the destination. A.1 and A.2) visualize results from the *multinomial logit* models that classify first moves into four network-defined categories (individual ties only, institutional ties only, both, or neither), plotted against the prestige of the source and target institutions, respectively. If the prestige of the source institution is low, scholars mostly move along individual ties or do not follow any connection. If it is high, however, scholars become more likely to move either along institutional connections or a combination of institutional and individual ties, and it becomes less likely for them to go somewhere where they have no connection yet. With regard to the target institution, if the prestige of the destination is low, scholars mostly move there without any previous connections. Target institutions of a high prestige ranking, on the contrary, are more frequently reached via institutional connections or a combination of institutional and individual ties compared to having no ties or having individual ties only. B.1–C.2) report results from the *destination-choice (conditional multinomial logit)* models, which relate the probability of selecting a specific destination institution to alternative-specific measures of (i) individual connection strength (B.1–B.2) and (ii) institutional connection strength (C.1–C.2), and their interactions with source and destination prestige. Strength percentiles are defined only for nonzero ties; accordingly, these panels condition on having at least one relevant connection. While we did not find meaningful heterogeneity in the effect of both the individual and institutional connection strength with regard to the prestige of the source institution (top row), we find clear heterogeneity with respect to the destination institution’s prestige (bottom row): institutions of lower prestige rank benefit from having ties to potential recruits more than institutions of higher prestige rank. This pattern is consistent across all levels of connection strengths for both individual and institutional connections, but gets more pronounced the stronger the connections are (see B.2 and C.2).

The prestige of both source and target institutions substantially shapes how connections influence migration decisions. Scholars affiliated with higher-ranked source institutions are more likely to follow institutional connections and less likely to rely on individual ties (see Fig. 3A.1), suggesting prestige homophily in hiring practices—a finding consistent with previous work (43). In addition, we did not find any differential effects in how connection strength (individual or institutional) operates based on the prestige of the source institution (see Figs. 3B.1 and C.1). The prestige of the target institution, however, exerts a more complex effect: The probability of moving along individual ties is associated with institutional rank, but peaks at medium-high ranks before declining at the highest levels. In contrast, the probability of moving along institutional pathways, or via a combination of both individual and institutional ties, rises steadily as the target institution’s prestige increases (see Fig. 3A.2). These findings suggest that moving *from* a high-prestige institution is commonly linked to institutional pathways, whereas moving *to* a high-prestige institution relies more on individual connections.

When examining how the effect of the connection strength varies by the prestige level of the target institutions, we find that lower-ranked institutions rely more heavily on both individual and institutional ties when recruiting scholars (see Fig 3B.2 and 3C.2). This effect becomes more pronounced as connection strength increases. In contrast, high-ranked institutions exhibit less reliance on preexisting ties, suggesting that other selection mechanisms, such as merit-based evaluation, play a stronger role in these contexts. Finally, when moves are classified as upward, lateral, or downward, no strong hierarchical asymmetry emerges, suggesting that relative prestige does not function as the primary organizing principle for early-career mobility (see Supplementary material Part 8, Fig. S3 and Table S12).

This result aligns with previously published arguments and findings: Institutional prestige can be viewed as a positional resource (location in a recognized status hierarchy) that shapes both supply and demand in academic labor markets. Highly prestigious institutions routinely attract a larger pool of high-quality applicants and can screen them on universally visible merit signals, such as citations, grants, and awards, which ultimately reduces dependence on personal referrals (35, 43). In contrast, mid- and lower-ranked institutions face higher information uncertainty and therefore have to rely more heavily on trusted channels, such as referrals from colleagues, to identify suitable hires (36). Furthermore, mobility along institutional pathways can be interpreted as being consistent with status-closure dynamics and prestige homophily, whereby high-status institutions preferably exchange talent among each other (34, 44). Our findings also echo a cumulative advantage logic: An early placement at a prestigious institution amplifies future opportunities independently of existing ties (43, 45).

### Other moderating factors

While the literature has discussed other personal and institutional characteristics which might also shape the direction of mobility decisions, their effects, in our analysis, tend to be less pronounced. Career age negatively correlates with network-directed migration—older scholars are more likely to fall into the “neither” category. This could mean that more senior scholars have accumulated enough research prestige through their prior work and publications that they could select destinations without prior ties. Productivity, measured by past publications, has a positive impact on moving along individual or a combination of both types of ties, indicating that more productive scholars are better positioned to leverage their networks. Gender differences are present but minor: men have a slightly lower probability of moving along individual ties, or a combination of both individual and institutional connections (see Supplementary material Part 4, Table S8). Destination-choice (conditional multinomial logit) analysis confirms that the direction of migration decisions is strongly shaped by connection structures, particularly in combination with institutional prestige. Notably, we did not find any gender effect in the extent to which men and women use their resources (see Supplementary material Part 5, Table S10). However, we did not investigate whether men and women differ structurally in their resources, ie which institutions they are placed in/they are connected to. This question is still open for further investigation.

Overall, our findings underscore the intertwined roles of personal connections, institutional prestige, and migration context in shaping academic mobility.

## Discussion

High-skilled labor migration is often framed as a rational response to institutional incentives or career advancement opportunities. Yet, the decision of where to move is deeply embedded in social structures. In this study, we focus on the first career moves of scholars, using digital trace data from 172,000 scholars that captures their complete mobility trajectories, co-authorship networks, and collaboration histories. We examine how individual and institutional connections and institutional prestige shape the first academic mobility. Using two complementary logit specifications for classifying move types and modeling destination choice, we document how professional connections jointly structure researchers’ opportunity spaces in their first career moves. The methodological approach adopted here, ie the use of large-scale bibliometric data to track migration and map relational structures, enables us to quantify these dynamics in ways that traditional migration data sources, such as surveys, cannot.

Rather than treating ties in isolation or combining different sets of ties without proper differentiation, we demonstrate that individual and institutional professional networks play distinct yet interconnected roles in shaping scholarly migration. We show that individual professional networks, and especially higher-order connections, are more strongly associated with where scholars move than institutional connections, although both matter, especially when they operate together. For example, scholars with large individual networks are more likely to follow individual connections, while those with smaller networks often move outside predefined career pathways. This suggests that professional individual connections not only provide opportunities but also enhance agency in mobility decisions. Additionally, international moves often occur outside established networks, with individual ties being particularly salient in cross-border transitions. This highlights the challenges of international mobility and the importance of personal connections in overcoming them, which is consistent with longstanding insights from migration literature (15). In contrast, national moves are more likely to follow institutional connections or combined ties, potentially amplifying within-country stratification.

We further find that institutional prestige moderates how a scholar’s embedding is associated with mobility. Researchers affiliated with high-ranking institutions are more likely to follow institutional pathways or a combination of institutional and individual-level professional ties, reflecting prestige homophily (43). Conversely, scholars affiliated with lower-ranked institutions tend to depend more on individual professional networks when making mobility decisions. Our results also show that prestigious institutions are able to attract researchers with less reliance on preexisting individual ties, while institutions with lower prestige benefit disproportionately more from strong individual-level connections during recruitment. This asymmetry underscores how institutional reputation interacts with social networks in structuring the first academic mobility.

While this study advances our understanding of how networks shape the migration of researchers, and their first career moves, several limitations must be acknowledged. First, scholarly migration represents a specific form of high-skilled mobility, and the role of networks may differ in other migration contexts. Second, while Scopus provides extensive data (46), its temporal and spatial coverage is skewed towards Western countries and English-language publications. Third, co-authorship networks may not capture all personal connections (40, 47), potentially underestimating the influence of individual ties. Fourth, our analyses are conditional on observing a first career move and therefore exclude early-career researchers who remain immobile or exit academia before a first observable transition. Because nonmobility is selective, and may be more common among scholars with more limited professional networks (26), our results may overrepresent network-mediated mobility pathways and should not be generalized to all early-career researchers. At the same time, we find that after adjusting for observable characteristics, movers and nonmovers do not differ significantly in the size of their co-authorship networks (see Supplementary material Part 7, Fig. S2). This suggests that nonmobility is not simply a function of having smaller professional networks, partially mitigating concerns that our estimates systematically overrepresent network-endowed scholars. Fifth, while we investigate gender differences in the use of ties, we do not address structural disparities in access to resources. Finally, our models treat mobility as a scholar-driven decision, whereas academic hiring is a two-sided matching process, involving both applicant and institution. Future work should integrate employer demand, for instance, by combining bibliometric mobility traces with job postings or department growth metrics.

Despite these limitations, we argue that our study advances our understanding of the social foundations of scientific mobility by showing how different types of network ties interact to shape scholars’ opportunity spaces. Our findings reaffirm that mobility decisions are not made in isolation but are fundamentally shaped by the relationships scholars form within the academic system (15, 17) as well as the reputation of the institutions they interact with. We propose several directions for future research, namely: (i) integrating a first-stage “move vs. stay” decision to distinguish between anchoring effects and steering effects of networks, (ii) exploring structural disparities in access to resources across genders, (iii) modeling the two-sided nature of academic hiring more explicitly, and (iv) examining the role of digital platforms in shaping mobility opportunities. By deepening our understanding of these dynamics, we can better support global scientific exchange and foster a more equitable and efficient allocation of talent in academia.

## Data and methods

### Data

For this study, we utilize a 2021 extract of Scopus, covering all records until the end of 2020 (46), a widely recognized data source for scholarly migration studies (11, 12, 48). These data are provided by the German Competence Network for Bibliometrics (49) via the Max Planck Digital Library. Our dataset includes around 172,000 authors (see Supplementary material Part 1). We identify mobility events using a cautious “mode-based” approach that reduces all affiliations recorded within a given year to the most frequent one, logging a mobility event only when this modal affiliation changes across successive years (11). Authors are included if sufficient affiliation information exists to reliably identify a mobility event, which in the minimum cases is two publications with different affiliations observed in two different years. To achieve this, we geocode affiliation addresses to subnational regions, enabling us to distinguish between internal (within a country) and international (between countries) mobility events (12). Authors are grouped by the year of first publication, which we term “academic cohorts”. These cohorts act as a proxy for entry into the academic system and starting publication activity. In our sample, we construct five nonoverlapping cohorts, each spanning five consecutive years, covering the full 1996–2020 observation period. The 5-year span ensures equally spaced intervals with sufficient observations per cohort. Importantly, these cohorts are used as control variables in the multinomial logit models to account for cohort effects, such as differences in publication frequency across different periods. They are not used for selecting subset of the authors as a sample, for aggregating transitions, or for constructing predictors. By taking these steps, we are able to construct complete individual-level mobility histories for each author. In addition to the mobility histories, we create authorship records, including authors’ first- and second-order co-authorship networks, publication histories, the country of an author’s affiliation, as well as their discipline, which is inferred based on the discipline classification of the publication using Scopus’ All Science Journal Classifications (46). We assign a discipline with the highest share of publications as the main one for the focal author. An author’s gender is inferred using their first name, provided by Ref. (48). As we are interested in studying how researchers’ network embedding influences the direction of their move, we focus on mobile authors only. Furthermore, we focus on first mobility events only (the mobility step after an author’s first publication, which is usually their first position after obtaining a PhD), as this stage is often especially critical for career trajectories and long-term outcomes (10, 38).

To measure individual connections, we use one of the most widely applied proxies for it: co-authorship ties. However, we acknowledge that this proxy only partially captures our interest in individual connections, as not all professional collaborative ties are reflected in the form of shared publications (40, 47). Hence, we expect our findings to be more on the conservative side. For each migrant scholar—those who moved at least once—we calculate the number of co-authors they had before their move, as well as the number of second-order ties, which represent the co-authors of their co-authors. This distinction is crucial, as it allows us to differentiate between the influence of direct connections and “friend of a friend” effects. For institutional pathways, we aggregate all co-authorships and migration events between research institutions. Using these distinct measures for both individual and institutional networks, we categorize the direction of movements based on whether the scholar’s move followed individual or institutional ties, resulting in four possible directions, namely: along individual ties only, along institutional ties only, along a combination of individual and institutional ties (referred to as “both”), and not along any ties (referred to as “neither”). We then test these categories using different models. Figure 1 shows a schematic visualization of these individual and institutional ties before the mobility event and our retrospective approach in measuring collaboration and mobility.

We use institutional rankings as a proxy for prestige, considering it as a potential factor influencing migration decisions (31, 32). Specifically, we use the Leiden Ranking’s^a^ metric for P(top 50 publications, which captures the number of publications ranked among the top 50% most frequently cited within the same field and publication year. This metric encompasses ∼1,400 universities from 2006 to 2018 (42). To account for individual-level characteristics that may also shape the direction of mobility, our models include individual characteristics—gender, career age at the time of migration (measured as the difference between the year of migration and the year of a scholar’s first publication in our database), academic field (following the Scopus All Science Journal Classification), and productivity (measured by the cumulative number of papers published before the migration event)—alongside network size as control variable (see the Table S1). We further account for contextual factors, namely whether a move was national or international, the individual’s academic cohort, the year of the move, and the continent of the source and target institutions.

As the predictors in our observational data vary endogenously only, and we cannot rule out all potential unobserved confounders, such as individual talent or quality of individuals’ research output, we interpret the estimated associations descriptively rather than causally.

### Strength measures

In our study, we aim to determine whether an individual (a scholar) moves along an individual direction—that is, influenced by their co-authorship connections—or along an institutional direction, shaped by broader affiliations between institutions. We condition on the event of a move, as we are interested in how a scholar’s embedding influences the direction of a move, and not whether it triggers a move in the first place.

Co-authorship-documented ties are a conservative proxy for scholars’ broader professional networks—just the visible tip of a much larger iceberg. Survey evidence and previous literature shows that many mobility-relevant relationships—eg mentor sponsorship, previous colleagues and acquaintances, informal conference contacts—never culminate in joint papers, while some multiauthor papers mask only peripheral interaction (40, 41). We therefore treat co-authorship as necessary but not sufficient evidence of professional connection, and interpret our coefficients as lower-bound estimates of network embeddedness. In the following, we describe how we operationalize these two dimensions to quantify the strength of these connections.

#### Constructing the individual-level tie strength measure

To quantify the individual-level embedding of an author before a mobility event occurs at T+1, we propose the individual-based strength measure, $S_{Fj}^{\mathrm{ind}}(T)$, specified in (Eq. 1). It describes how strongly an individual *F* is connected to an institution *j* via their first- and second-order co-authorship network in the years before the mobility event (cumulative until *T*). If an individual held several institutional affiliations at any given year, we assign the modal affiliation of that year. Furthermore, each co-author (first- and second-order) is counted only once, even if published with repeatedly. This choice focuses the measure on access to distinct social contacts rather than repeated interaction intensity. It is defined as follows:


$$S_{Fj}^{\mathrm{ind}}(T)=\frac{C_{Fj}^{1\mathrm{stand}2\mathrm{nd}}(T)}{\sum_{\;j}C_{Fj}^{1\mathrm{stand}2\mathrm{nd}}(T)},$$

where $C_{Fj}^{1\mathrm{stand}2\mathrm{nd}}$ represents the number of first- and second-order co-authors the individual *F* has at institution *j* at time *T*, and $\sum_{\;j}C_{Fj}^{1\mathrm{stand}2\mathrm{nd}}$ is the total number of first- and second-order co-authors the individual *F* has at the same time point *T*, regardless of their institution.

Note that $S_{Fj}^{\mathrm{ind}}(T)$ is the proportion (share) of a scholar’s (first- and second-order) co-author network located at institution *j*. The measure is bound between 0 and 1; it captures relative embeddedness rather than the absolute number of co-authors.

A consequence of this definition is that two cases with the same share (eg 1 of 2 co-authors at *j* vs. 50 of 100 at *j*) will have the same connection strength $S_{Fj}^{\mathrm{ind}}(T)$; differences in absolute network size are captured separately by our network-size controls.

We also examined the observed effects when only first-order connections were considered. The respective formula can be found in the Supplementary material Part 3 (Eq.1).

#### Constructing the institutional-level connection measure

To quantify the institutional-level connection strengths, we propose a time-dependent measure based on past collaborations. The connection strength is evaluated over the 5 years preceding the year of the migration event. Two institutions are considered linked if they co-authored an article in a given year. The collaboration-based strength measure, $S_{ij}^{\mathrm{inst}}(T)$, between institution *i* and institution *j* at a given year *T* takes the following form (Eq. 2):


$$S_{ij}^{\mathrm{inst}}(T)=\sum_{t=T-5}^{T}\frac{A_{ij}(t)}{\sum_{k,k\neq i}A_{ik}(t)},$$

where $A_{ij}(t)$ is the total number of articles co-authored by scholars at the two institutions *i* and *j* at time *t* and $\sum_{k}A_{ik}(t)$ is the total number of articles co-authored by scholars at any other institution *k* in that year. This proportional construction makes institutional strengths comparable across institutions of different sizes. Note that the two strength measures (individual level and institutional level) differ by design: at the individual level we count unique collaborators (each co-author once), whereas at the institutional level we weight ties by the number of co-authored publications.

Because institutional collaboration networks are substantially denser than individual co-authorship networks, we operationalize the *presence* of an institutional tie differently depending on the analysis. For the decision-tree classification and for constructing the four-category move-type outcome, we therefore operationalize the presence of an institutional tie using a threshold indicator: a move is classified as “along an institutional tie” only if the source–destination institutional collaboration strength falls within the top 10% of nonzero institutional connections from the scholar’s prior institution at the time of the move. This threshold is used only for defining move categories; the multinomial logit model is estimated on the resulting four-category outcome.

In the destination-choice analysis (estimated as a conditional multinomial logit model), we instead use a continuous institutional-strength measure to capture gradations in institutional embeddedness. To improve computational efficiency, we implement a period-specific version of $S_{ij}^{\mathrm{inst}}(T)$ (as stated in Eq. 2): rather than a rolling window, we compute institutional connection strength within discrete time intervals (pre-2000, 2000–2005, 2005–2010, 2010–2015, and post-2015) and by field (Eq. 3).


$$S_{ij}^{{\mathrm{inst}}_{\mathrm{DCM}}}(\Delta T)=\frac{A_{ij}^{\mathrm{field}}(\Delta T)}{\sum_{k,k\neq i}A_{ik}^{\mathrm{field}}(\Delta T)}.$$

We use the top-10% indicator $S_{ij}^{\mathrm{inst}}(T)$ (based on Eq. 2) to define institutional tie presence; conditional on presence, institutional strength is measured using $S_{ij}^{{\mathrm{inst}}_{\mathrm{DCM}}}(\Delta T)$ (Eq. 3).

As a robustness check, we also tested an alternative specification for the institutional strength measure, considering past mobility between institutions instead of past collaborations (see Supplementary material Parts 3 and 4, Tables S5, S6, S7, and S9). The corresponding formula can be found in the Supplementary material Part 3 (Eq. 2). To assess potential multicollinearity among key predictors, we examined pairwise Spearman correlations; productivity and network size exhibit the strongest association (ρ=0.51), with all other correlations remaining modest in magnitude (see Supplementary material S6, Table S11).

### Modeling strategies

In the first set of analyses, we estimate multinomial logit models with a four-category outcome that classifies each first move based on its context: moves along individual ties, along institutional ties, along both, or with no prior tie. The model estimates the probability that a scholar’s move falls into each category. Because the four categories are defined by the existence of at least one prior tie to the destination, scholars with larger networks are mechanically more likely to have destinations that qualify as “along ties.” Thus, network size reflects both structural exposure (ie the likelihood that a tied destination exists) and potential selection along existing connections.

In the second set of analyses, we estimate destination-choice models in a conditional multinomial logit (discrete-choice) framework, shifting the focus from move types to how individual and institutional connection strengths are associated with the probability of selecting a particular destination institution.

#### Multinomial logit model for move types

To analyze how individual and contextual factors are associated with the type of first mobility event, we estimate a multinomial logit model with four mutually exclusive categories: moves along individual ties, along institutional ties, along both, or with no prior tie. The model compares each category to a reference category and expresses effects as log-odds ratios relative to that reference. For a model with *c* categories and *p* explanatory variables, the logit for category *j*, relative to the baseline category c,j≠c, can be expressed as (Eq. 4):


$$\log\;\left(\frac{\pi_{j}}{\pi_{c}}\right)=\alpha_{j}+\beta_{\;j1}x_{1}+\beta_{\;j2}x_{2}+\cdots +\beta_{\;jp}x_{p},\;j=1,\ldots ,c-1.$$

In this form, $\alpha_{j}$ represents the intercept for category *j*, and $\beta_{\;jk}$ denotes the effect of the *k*th explanatory variable $x_{k}$ on the log-odds of being in category *j* rather than the baseline. Each category thus has distinct parameter values, allowing flexible modeling of varied influences across categories.

The set of control variables includes the following personal characteristics: (i) gender, (ii) career age at the time of migration (measured as the difference between the year of migration and the year of a scholar’s first publication in our database), (iii) academic field, (iv) productivity (measured by the total number of papers published before migration), and size of the network (measured by the size of the first- and second-order co-author network). Productivity and network size are stratified into tertiles and quantiles within each academic field, respectively, to account for variations in publishing behavior. For the size of the network, those in the first quantile are classified as having small networks, those between the second and third quantiles as having medium-sized networks, and those in the top quantile as having large networks. We also include contextual variables to account for the environment in which migration decisions are made (50). These variables include (v) whether a move was national or international, (vi) the individual’s academic cohort (determined by the year of their first observed publication), (vii) the timing of the move (the time period in which the move happened), (viii) the region of the source and target institutions, and, importantly, (ix) the prestige rankings of both source and target institutions.

#### Destination-choice model (conditional multinomial logit)

While the move-type analysis can provide insights into the likelihood of a scholar with certain characteristics (gender, career age, etc.) exhibiting a particular type of move (along individual ties, institutional ties, along ties of both kind, or without ties), the question of how much the actual connection strength matters for an individual’s decision-making process, and whether individual or institutional ties more strongly shape the opportunity space for scholars, remains unresolved.

To address these questions, we formulate the analysis within the framework of discrete choice analysis (51, 52), estimated as a conditional multinomial logit model: Here, the decision-making process of every author is modeled at the individual level, taking the characteristics of the alternatives as well as the individual into account. This allows us to model the connection strengths as independent variables that influence the choice process of an author. In this modeling framework, authors are presented with different institutions they can choose from—hypothetical ones and the actually chosen ones. These institutions differ in their attributes, in our case, the individual and institutional connection strengths, as well as the prestige ranking. The probability that an individual scholar *i* chooses to move to an institution *j* is then given by Eq. 5:


$$Pr(y_{ij}=1)=\frac{\exp\;\left(\beta^{⊤}X_{ij}-\log\;(q_{ij})\right)}{\sum_{(j^{\prime })\in C_{i}}\exp\;\left(\beta^{⊤}X_{ij^{\prime }}-\log\;(q_{ij^{\prime }})\right)}\;$$

where $X_{ij}$ is a matrix of predictors that describe an individual *i*’s attributes as well as alternative-level attributes for the institution *j* as seen by the individual *i*. The index *j*′ denotes each possible destination institution in individual *i*'s choice set $c_{i}$, over which the denominator sums.

In the destination-choice analysis, we included the following variables: (i) a binary indicator for whether an individual has at least one individual-level tie to a potential target institution, (ii) a binary indicator for whether an alternative is among the top 10% of institutions in terms of collaborations based on Eq. 2, (iii) a continuous indicator, that given (i), indicates the strength of the connection as specified in Eq. 1, (iv) a continuous indicator, that given (ii), indicates the strength of the connection as specified in Eq. 3, (v) the prestige ranking of the target institution, (vi) the prestige ranking of the source institution, (viii) whether a move is international or national, and (viii) in which region of the world an alternative is located. We furthermore included interaction terms between the different strength measures and the ranking of the source and target institution to check for heterogeneity in the effect of existing ties, ie between (i) and (v), (i) and (vi), (ii) and (v), (ii) and (vi), (iii) and (v), (iii) and (vi), (iv) and (v), and (iv) and (vi). *j* indexes the alternatives present in individual *i*’s choice set.

Because vacancy data are unavailable, we make an assumption about which alternatives a scholar realistically could have had available as options. For our specific case, we used a stratified sampling approach to generate choice sets of size 50. The four mutually exclusive sets of institutions from which we sampled correspond to the categories used to classify mobility events in the decision tree and multinomial logistic regression framework, namely: institutions that an individual was connected to via (i) both institutional and individual level ties (sampled at 20%), (ii) institutional level ties only (35%), (iii) individual-level ties only (35%), or (iv) neither tie (10%, or as much was needed to fill the remaining open spots). This stratified sampling approach implies that choice sets vary across individual decision-makers. As the alternatives present in the choice set were not chosen randomly, we included an adjustment parameter in the model to account for their uneven likelihoods of inclusion: $q_{ij}$, where $q_{ij}$ represents the probability of sampling alternative *j* into the choice set of respondent *i* (53, 54).

Sampling a choice set using prior knowledge can improve the precision of the estimates, as the choice sets will exhibit more of the alternatives that an individual is *a priori* more likely to choose, rather than noise from alternatives which are unlikely to be selected. As most institutions in the total set of institutions are not connected to an individual of interest, ie having a connection is a rare event, weighted sampling ensures more realistic choice sets (51). The marginal probability plots were produced by constructing a prediction dataset using a sample of the individuals who were presented with the full choice set (all possible institutions). Note that the plotted marginal predicted relative probabilities should not be interpreted in absolute terms: They are meaningful only when interpreted relative to each other.

## Supplementary Material

pgag168_Supplementary_Data

## Data Availability

The raw and processed data at the individual level are not available due to Scopus license terms. Interested researchers can reach out to Elsevier/Scopus on accessing these data. Data and scripts to replicate the figures in the manuscript are available on GitHub (https://github.com/OlaAM/Scholarly_Migration/tree/main) and archived on OSF (https://doi.org/10.17605/OSF.IO/EVDHU).

## Notes

a
https://www.leidenranking.com/

## Preprints

This manuscript was posted on a preprint: https://dx.doi.org/10.4054/MPIDR-WP-2025-028.
