# Supplementary material for: The role of professional networks and institutional prestige in shaping the first career moves of scholars
Source: PNAS Nexus. 2026 May 15;5(6):pgag168. doi: 10.1093/pnasnexus/pgag168 (PMC13224738; doi:10.1093/pnasnexus/pgag168)
Supplement: pgag168_Supplementary_Data [file pgag168_supplementary_data.pdf]

PNAS nexus

Supporting Information for

The Role of Professional Networks and Institutional Prestige in Shaping the First Career Moves of Scholars

Alexandra Rottenkolber, Ola Ali, Gergely Mónus, Jiaxuan Li, Jisu Kim, Daniela Perrotta, and Aliakbar Akbaritabar

Aliakbar Akbaritabar
E-mail: akbaritabar@demogr.mpg.de

This PDF file includes:

Figs. S1 to S3
Tables S1 to S12
SI References

1. Descriptive view of the data

Table S1 summarizes key characteristics of the study sample comprising 171,746 mobile scholars identified using Scopus-based bibliometric data. On average, individuals had published approximately 10 papers (SD = 20.51) and collaborated with over 15 first-order coauthors and more than 700 first- and second-order coauthors before their first move. The mean academic age at migration was 5.9 years (SD = 3.86). Institutional prestige, measured via the Leiden Ranking*, was broadly distributed, with average target and source institution rankings around 3,800. The sample is skewed towards male scholars (66.7%) and is largely concentrated in Physical Sciences (44.6%), followed by Life Sciences (24.4%) and Health Sciences (19.7%), which is similar to the overall composition of scholars across fields of science in Scopus data (1). Most mobility events were national (55.3%), although international moves were also common (44.7%). The sample also spans a wide range of academic cohorts from 1996 to 2020, allowing to capture temporal variation in mobility patterns. Strength measures indicate that approximately 47.2% of scholars moved along at least one individual-level tie, while 46.0% moved along an institutional tie. Notably, only 19.1% had strong first-order-only individual connections, and 22.7% moved along alternative institutional pathways.

Variable	Overall (N=171,746)
**Cumulative Number of Publications**	9.97 (20.51)
**Number of First Order Coauthors**	15.36 (44.05)
**Number of First and Second Order Coauthors**	725.90 (2417.14)
**Career Age (Years)**	5.90 (3.86)
**Target Institution Ranking**	3,815.43 (3442.37)
**Source Institution Ranking**	3,908.10 (3307.17)
**Individual connection strength, continuous**	0.03 (0.08)
**Institutional connection strength, continuous**	0.01 (0.03)
**Gender**	
Female	57,185 (33.3%)
Male	114,561 (66.7%)
**Academic Cohort**	
1996-2000	24,514 (14.3%)
2001-2005	43,065 (25.1%)
2006-2010	59,687 (34.8%)
2011-2015	41,897 (24.4%)
2016-2020	2,583 (1.5%)
**ASJC Classification (Top)**	
Health Sciences	33,844 (19.7%)
Life Sciences	41,836 (24.4%)
Physical Sciences	76,665 (44.6%)
Social Sciences	19,109 (11.1%)
Unknown	292 (0.2%)
**Move Type**	
International move	76,814 (44.7%)
National move	94,932 (55.3%)
**Strength Measures**	
$S^{\text{ind}_{alt}}_{Fj}(T)$ Binary = 1	32,861 (19.1%)
$S^{\text{ind}}_{Fj}(T)$ Binary = 1	81,088 (47.2%)
$S^{\text{inst}}_{ij}(T)$ Binary = 1	79,080 (46.0%)
$S^{\text{inst}_{alt}}_{ij}(T)$ Binary = 1	38,985 (22.7%)

Table S1. Descriptive Statistics of the Study Sample

2. Decision Tree Results

We constructed a decision tree to analyze the direction of scholarly mobility, classifying each move into one of four categories: along individual ties, along institutional ties, along both, or along neither. The model incorporates individual-level features (e.g., gender, career age, and connectedness) as well as contextual characteristics (e.g., move type and institutional rankings) to predict the direction of a scholar's movement. Our primary aim with this model was predictive—to identify the most important features that influence mobility direction.

* https://www.leidenranking.com/

We trained the model using the `DecisionTreeClassifier` from Python's `sklearn.tree` package (2). The decision tree offers a transparent and interpretable framework, revealing both the hierarchy and relative importance of input variables. Unlike more complex models, its structure allows for a direct understanding of how specific features influence the classification outcome, i.e., the type of the move.

The dataset was randomly split into a 70% training set and a 30% test set. The classifier was configured with `criterion= entropy`, `max_depth=10`, and `min_samples_leaf=10`, and class weights were balanced to mitigate class imbalance (Table S2). The model achieved a weighted F1-score between 0.66 and 0.74 across categories (Table S4), and an overall accuracy between 0.72 and 0.77 (Table S3), indicating a solid and balanced performance across the four mobility pathways.

Among all input features, the size of a scholar's second-order network—measured by the number of coauthors and co-coauthors—emerged as the most influential predictor, contributing approximately 40% to the model's decision-making process (Figure S1). This finding underscores the critical role of extended personal networks in shaping mobility patterns, suggesting that indirect connections (i.e., "friends-of-friends") may be particularly associated with the moves.

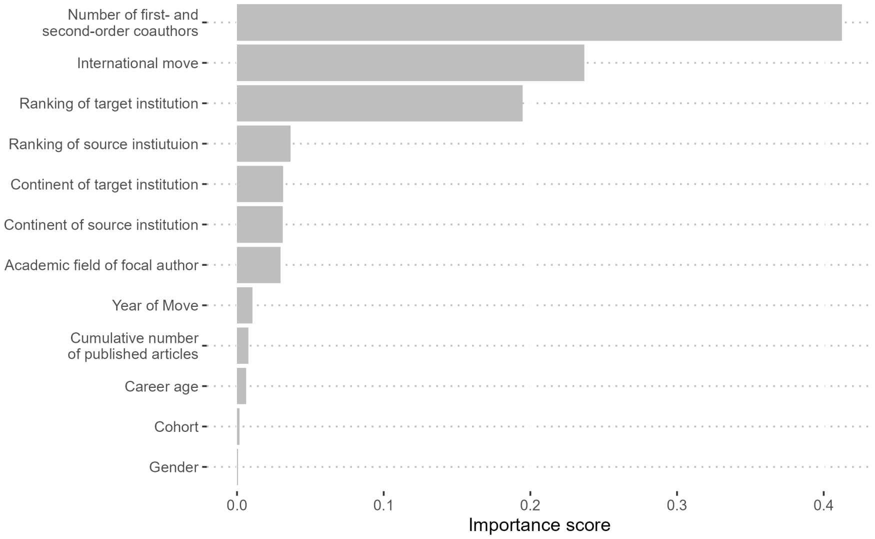

Fig. S1. Decision tree. The higher the importance score the more predictive a variable is for the type of mobility event observed (whether a move followed individual ties only, institutional ties only, both, or none of them).

The next most important features were the type of move (national vs. international) and the ranking of the target institution. The importance of move type supports the idea that international mobility often follows different mechanisms than domestic moves—potentially relying more on individual-level connections and less on institutional pipelines. Likewise, the influence of institutional rankings suggests that prestige considerations weigh heavily in scholars' decision-making processes, potentially guiding them toward highly ranked destinations when such opportunities are available.

Parameter	Value
class_weight	balanced
criterion	entropy
max_depth	10
max_features	None
min_samples_leaf	10
min_samples_split	2

Table S2. Decision Tree Parameters

3. Alternative operationalization of the strength measures

Acknowledging that past collaborations only serve as a limited proxy for individual and institutional connections (3, 4), we tested two additional measures to quantify individual and institutional connection strengths.

Direction	Accuracy
Following Individual Direction	0.72
Following Institutional Direction	0.74
Not Following Either Direction	0.74
Following Both Direction	0.77

Table S3. Accuracy of the decision tree in prediction of each direction

Direction	Precision	Recall	F1-score	Support
Following Individual Direction	0.70	0.75	0.72	24458
Following Institutional Direction	0.70	0.77	0.74	23760
Not Following Either Direction	0.64	0.67	0.65	18060
Following Both Direction	0.58	0.76	0.66	14723
Micro avg	0.66	0.74	0.70	81001
Macro avg	0.65	0.74	0.69	81001
Weighted avg	0.66	0.74	0.70	81001
Samples avg	0.65	0.69	0.63	81001

Table S4. Classification Report of the decision tree for each direction

A. Alternative individual-level tie strength measure. For the individual level metric, we also evaluated our analysis when taking only first-order ties into account. The corresponding measure $S_{Fj}^{\text{ind}_{alt}}(T)$, specified in Eq. 1, quantifies how strongly an individual F is connected to an institution j via their first-order co-authorship network in the year before a mobility event. It is defined as follows:

$$S_{Fj}^{\text{ind}_{alt}}(T) = \frac{C_{Fj}^{\text{1st}}(T)}{\sum_j C_{Fj}^{\text{1st}}(T)} \quad [1]$$

where C_{Fj}^{1st} represents the number of first-order co-authors that individual F has at institution j at time T and $\sum_j C_{Fj}^{\text{1st}}$ is the total number of first-order co-authors that individual F has at the same time point T, the year before the mobility event, regardless of their institution.

B. Alternative institutional-level tie strength measure. The alternative measure we tested for the strength of the institutional connection is based on the past mobility of scholars between institutions. Specifically, two institutions are considered linked if individuals migrated from one to the other in a given year. The mobility-based strength measure, $S_{ij}^{\text{inst}_{alt}}(T)$, between institution i and institution j at a given year T takes the following form (Eq.2):

$$S_{ij}^{\text{inst}_{alt}}(T) = \sum_{t=T-5}^{T} \frac{M_{ij}(t)}{\sum_{j \neq i} M_{ij}(t)} \quad [2]$$

where $M_{ij}(t)$ is the total number of individuals migrating between the two institutions and $\sum_j M_{ij}(t)$ is the total number of individuals who migrated from or to institution i, excluded those who migrated from or to institution j.

C. Comparison of measures. The two measures for the institutional level, where one is based on past collaborations and one on past mobility events, could be considered different proxies for the influence an institution has on the individual migration direction. For the main analysis, we took $S_{ij}^{\text{Inst}_{alt}}$ as it covers more percentage of the individuals (Table S5).

	Individual-level connection strength		Institutional-level connection strength	
Strength measure	$S_{Fj}^{\text{ind}_{alt}}$	S_{Fj}^{ind}	S_{ij}^{inst}	$S_{ij}^{\text{inst}_{alt}}$
% of individuals	22.0%	45.0%	35.0%	18.7%

Table S5. Percentages of individuals who moved along a certain connection.

4. Multinominal Logistic Regression Models

As a robustness measure, we fit our network direction-based multinomial logistic regression models with all possible measurement combinations. There are four models all together, combining the two main measure and two alternative measure. Our results show that while effect sizes vary between the different models, the significances and directions are consistent across them.

Table S6. Regression table - Multinominal logistic regressions - Individual direction measured with first-order coauthorship connections ($S_{Fj}^{\text{ind}_{alt}}$), institutional direction measured with aggregated past coauthorships between institutions (S_{Fj}^{inst})

	Dependent variable:		
	both	individual_direction	institutional_direction
	(1)	(2)	(3)
Gender (male == TRUE)	−0.027	-0.049^{**}	−0.010
	(0.020)	(0.021)	(0.014)
National move (TRUE)	2.357^{***}	-0.146^{***}	2.403^{***}
	(0.023)	(0.022)	(0.016)
Career Age	−0.008	-0.031^{***}	−0.004
	(0.005)	(0.005)	(0.004)
Network size (Medium)	0.793^{***}	0.679^{***}	0.051^{***}
	(0.028)	(0.027)	(0.016)
Network size (High)	1.607^{***}	1.349^{***}	0.070^{***}
	(0.030)	(0.030)	(0.020)
Productivity (Medium)	0.109^{***}	0.150^{***}	−0.022
	(0.026)	(0.027)	(0.016)
Productivity (High)	0.508^{***}	0.524^{***}	-0.034^{*}
	(0.029)	(0.030)	(0.021)
log(Ranking target institution)	0.972^{***}	0.090^{***}	0.962^{***}
	(0.011)	(0.010)	(0.008)
log(Ranking source institution)	0.255^{***}	-0.046^{***}	0.427^{***}
	(0.011)	(0.010)	(0.008)
Cohort fixed effects	Included	Included	Included
Period fixed effects	Included	Included	Included
Field fixed effects	Included	Included	Included
Geographic controls	Included	Included	Included
Constant	-10.089^{***}	-1.696^{***}	-9.938^{***}
	(0.155)	(0.151)	(0.114)
Akaike Inf. Crit.	322,959.6	322,959.6	322,959.6

Notes: *p<0.1; **p<0.05; ***p<0.01. Cohort fixed effects include indicators for cohorts 2001-2005, 2006-2010, 2011-2015, and 2016-2020. Period fixed effects include indicators for move periods 2011-2015 and 2016-2020. Field fixed effects include indicators for Life Sciences, Physical Sciences, Social Sciences, and other fields. Geographic controls include indicators for current and previous continents (Asia, Australia, Europe, Europe/Asia, North America, and South America).

Table S7. Regression table - Multinominal logistic regressions - Individual direction measured with first-order coauthorship connections ($S_{Fj}^{\textbf{ind}_{alt}}$), institutional direction measured with aggregated past mobility of scholars between institutions ($S_{Fj}^{\textbf{inst}_{alt}}$)

	Dependent variable:		
	both	individual_direction	institutional_direction
	(1)	(2)	(3)
Gender (male == TRUE)	0.014	-0.035^{**}	0.032^{**}
	(0.025)	(0.016)	(0.016)
National move (TRUE)	2.742^{***}	-0.065^{***}	2.687^{***}
	(0.036)	(0.017)	(0.022)
Career age	-0.007	-0.025^{***}	-0.014^{***}
	(0.007)	(0.004)	(0.004)
Network size (Medium)	0.735^{***}	0.726^{***}	-0.019
	(0.036)	(0.023)	(0.019)
Network size (High)	1.528^{***}	1.457^{***}	-0.025
	(0.039)	(0.024)	(0.024)
Productivity (Medium)	0.150^{***}	0.129^{***}	-0.020
	(0.034)	(0.022)	(0.019)
Productivity (High)	0.412^{***}	0.541^{***}	-0.129^{***}
	(0.038)	(0.024)	(0.025)
log(Ranking target institution)	0.935^{***}	0.088^{***}	0.907^{***}
	(0.014)	(0.008)	(0.009)
log(Ranking source institution)	0.376^{***}	-0.083^{***}	0.573^{***}
	(0.014)	(0.008)	(0.009)
Cohort fixed effects	Included	Included	Included
Period fixed effects	Included	Included	Included
Field fixed effects	Included	Included	Included
Geographic controls	Included	Included	Included
Constant	-12.813^{***}	-1.408^{***}	-12.449^{***}
	(0.206)	(0.122)	(0.138)
Akaike Inf. Crit.	285,709.6	285,709.6	285,709.6

Notes: *p<0.1; **p<0.05; ***p<0.01. Cohort fixed effects include indicators for cohorts 2001-2005, 2006-2010, 2011-2015, and 2016-2020. Period fixed effects include indicators for move periods 2011-2015 and 2016-2020. Field fixed effects include indicators for Life Sciences, Physical Sciences, Social Sciences, and other fields. Geographic controls include indicators for current and previous continents (Asia, Australia, Europe, Europe/Asia, North America, and South America).

Table S8. Regression table - Multinominal logistic regressions - Individual direction measured with first- and second-order coauthorship connections (S^{ind}_{Fj}), institutional direction measured with aggregated past coauthorships between institutions (S^{inst}_{Fj})

	Dependent variable:		
	both	individual_direction	institutional_direction
	(1)	(2)	(3)
Gender (male == TRUE)	-0.050^{***}	-0.047^{***}	0.010
	(0.016)	(0.016)	(0.017)
National move (TRUE)	2.795^{***}	0.357^{***}	2.238^{***}
	(0.019)	(0.017)	(0.019)
Career age	-0.020^{***}	-0.022^{***}	-0.009^{**}
	(0.004)	(0.004)	(0.005)
Network size (Medium)	1.098^{***}	0.948^{***}	-0.174^{***}
	(0.020)	(0.020)	(0.019)
Network size (High)	2.057^{***}	1.877^{***}	-0.424^{***}
	(0.023)	(0.022)	(0.025)
Productivity (Medium)	0.182^{***}	0.261^{***}	-0.024
	(0.019)	(0.019)	(0.019)
Productivity (High)	0.527^{***}	0.622^{***}	-0.059^{**}
	(0.022)	(0.021)	(0.024)
log(Ranking target institution)	1.182^{***}	0.285^{***}	0.885^{***}
	(0.009)	(0.008)	(0.009)
log(Ranking source institution)	0.267^{***}	-0.098^{***}	0.452^{***}
	(0.009)	(0.008)	(0.009)
Cohort fixed effects	Included	Included	Included
Period fixed effects	Included	Included	Included
Field fixed effects	Included	Included	Included
Geographic controls	Included	Included	Included
Constant	-10.968^{***}	-2.317^{***}	-9.748^{***}
	(0.135)	(0.125)	(0.133)
Akaike Inf. Crit.	356,441.9	356,441.9	356,441.9

Notes: *p<0.1; **p<0.05; ***p<0.01. Cohort fixed effects include indicators for cohorts 2001-2005, 2006-2010, 2011-2015, and 2016-2020. Period fixed effects include indicators for move periods 2011-2015 and 2016-2020. Field fixed effects include indicators for Life Sciences, Physical Sciences, Social Sciences, and other fields. Geographic controls include indicators for current and previous continents (Asia, Australia, Europe, Europe/Asia, North America, and South America).

Table S9. Regression table - Multinominal logistic regressions - Individual direction measured with first- and second-order coauthorship connections (S^{ind}_{Fj}), institutional direction measured with aggregated past mobility of scholars between institutions ($S^{\text{inst}_{alt}}_{Fj}$)

	Dependent variable:		
	both	individual_direction	institutional_direction
	(1)	(2)	(3)
Gender (male == TRUE)	−0.015	-0.056^{***}	0.046^{**}
	(0.019)	(0.014)	(0.022)
National move (TRUE)	3.254^{***}	0.552^{***}	2.490^{***}
	(0.026)	(0.014)	(0.029)
Career age	-0.020^{***}	-0.022^{***}	-0.022^{***}
	(0.005)	(0.004)	(0.006)
Network size (Medium)	1.115^{***}	1.027^{***}	-0.236^{***}
	(0.024)	(0.016)	(0.024)
Network size (High)	2.062^{***}	2.042^{***}	-0.510^{***}
	(0.027)	(0.019)	(0.034)
Productivity (Medium)	0.197^{***}	0.247^{***}	−0.024
	(0.022)	(0.016)	(0.024)
Productivity (High)	0.432^{***}	0.625^{***}	-0.063^{**}
	(0.025)	(0.018)	(0.032)
log(Ranking target institution)	1.196^{***}	0.342^{***}	0.833^{***}
	(0.011)	(0.007)	(0.013)
log(Ranking source institution)	0.420^{***}	-0.110^{***}	0.566^{***}
	(0.011)	(0.007)	(0.013)
Cohort fixed effects	Included	Included	Included
Period fixed effects	Included	Included	Included
Field fixed effects	Included	Included	Included
Geographic controls	Included	Included	Included
Constant	-13.816^{***}	-2.416^{***}	-11.514^{***}
	(0.163)	(0.107)	(0.171)
Akaike Inf. Crit.	320,691.3	320,691.3	320,691.3

Notes: *p<0.1; **p<0.05; ***p<0.01. Cohort fixed effects include indicators for cohorts 2001-2005, 2006-2010, 2011-2015, and 2016-2020. Period fixed effects include indicators for move periods 2011-2015 and 2016-2020. Field fixed effects include indicators for Life Sciences, Physical Sciences, Social Sciences, and other fields. Geographic controls include indicators for current and previous continents (Asia, Australia, Europe, Europe/Asia, North America, and South America).

5. Discrete Choice Models

Regression table for the discrete choice model. We estimated three different models: (1) on the full data, (2) on male scholars only, and (3) on female scholars only. The latter two were performed to test for gender differences, but none were observed.

Table S10. Regression table

	Dependent variable:		
	choice	choice	choice
	(1) all	(2) men only	(3) women only
S^{ind}_{Fj} binary (TRUE)	−6.994***	−6.191***	−8.609***
	(0.398)	(0.487)	(0.695)
$S^{\text{inst}_{DCM}}_{ij}$ binary (TRUE)	−1.128***	−1.223***	−0.911**
	(0.247)	(0.298)	(0.440)
S^{ind}_{Fj} continuous	0.728***	0.669***	0.846***
	(0.027)	(0.033)	(0.047)
$S^{\text{inst}_{DCM}}_{ij}$ continuous	0.480***	0.477***	0.488***
	(0.018)	(0.022)	(0.033)
International move (TRUE)	0.569***	0.581***	0.524***
	(0.053)	(0.064)	(0.097)
log(Ranking target institution)	−0.385***	−0.421***	−0.327***
	(0.052)	(0.063)	(0.091)
Region (Asia)	−0.105***	−0.095***	−0.143***
	(0.025)	(0.031)	(0.046)
Region (Australia)	0.170***	0.109***	0.288***
	(0.028)	(0.034)	(0.049)
Region (Europe)	−0.245***	−0.303***	−0.129***
	(0.025)	(0.031)	(0.045)
Region (North America)	0.208***	0.160***	0.304***
	(0.025)	(0.031)	(0.046)
Region (South America)	−0.003	−0.106***	0.172***
	(0.030)	(0.037)	(0.052)
S^{ind}_{Fj} binary (TRUE):log(Ranking target institution)	0.101***	0.112**	0.086
	(0.038)	(0.046)	(0.066)
$S^{\text{inst}_{DCM}}_{ij}$ binary (TRUE):log(Ranking target institution)	−0.114***	−0.093***	−0.151***
	(0.020)	(0.024)	(0.036)
S^{ind}_{Fj} continuous:log(Ranking target institution)	−0.027***	−0.028***	−0.024***
	(0.003)	(0.003)	(0.005)
$S^{\text{inst}_{DCM}}_{ij}$ continuous:log(Ranking target institution)	−0.025***	−0.025***	−0.026***
	(0.002)	(0.002)	(0.003)
S^{ind}_{Fj} binary (TRUE):log(Ranking source institution)	−0.034	−0.145***	0.186**
	(0.042)	(0.052)	(0.073)
$S^{\text{inst}_{DCM}}_{ij}$ binary (TRUE):log(Ranking source institution)	0.058**	0.062**	0.042
	(0.024)	(0.029)	(0.043)
S^{ind}_{Fj} continuous:log(Ranking source institution)	0.011***	0.020***	−0.006
	(0.003)	(0.004)	(0.005)
$S^{\text{inst}_{DCM}}_{ij}$ continuous:log(Ranking source institution)	0.006***	0.005**	0.008***
	(0.002)	(0.002)	(0.003)
Observations	8,582,100	5,724,300	2,857,800
Log Likelihood	−482,282.900	−323,538.200	−158,550.700
LR Test (df = 19)	615,618.600***	401,570.700***	214,435.800***
Note:			*p<0.1; **p<0.05; ***p<0.01

6. Multicollinearity Diagnostics

To assess potential multicollinearity among the key predictors, we examined pairwise associations using a Spearman correlation matrix (Table S11). As expected in scientific career data, productivity and network size exhibit a moderate positive correlation ($\rho = 0.51$), reflecting the well-documented linkage between publication output and collaborations. This association is also mechanically consistent with our operationalization of individual connection strength in Equation (1): higher productivity increases the likelihood of co-authorship events, which in turn expands an author's collaboration network, either directly through a larger number of first-order ties or indirectly through second-order ties.

Correlations between prestige measures (log current and previous rankings) and the other predictors are positive but modest in magnitude, and the correlation between current and previous rankings remains well below levels typically associated with problematic multicollinearity.

	Career Age	Productivity	Network Size	log (Ranking of target institution)	log (Ranking of source institution)
Career Age	1	0.390^{***}	0.230^{***}	-0.028^{***}	0.022^{***}
Productivity	0.390^{***}	1	0.514^{***}	0.029^{***}	0.052^{***}
Network Size	0.230^{***}	0.514^{***}	1	0.149^{***}	0.159^{***}
log (Ranking of target institution)	-0.028^{***}	0.029^{***}	0.149^{***}	1	0.216^{***}
log (Ranking of source institution)	0.022^{***}	0.052^{***}	0.159^{***}	0.216^{***}	1

Table S11. Pairwise Spearman correlations between career age, productivity, collaboration network size, and log-transformed rankings of target and source institution.
*****p<0.01**

7. Matching Analysis: Co-authorship Network Size of Movers and Non-movers

To further assess whether the absence of a first mobility event is associated with differences in professional network size, we conducted an additional matching analysis comparing movers and non-movers with all observable characteristics available. Specifically, non-movers are defined as authors for whom no change in institutional affiliation was observed during the corresponding observation window following their first publication. To reduce imbalance in observable characteristics, we implemented a 1:1 nearest-neighbour matching procedure without replacement. Each mover was matched to one non-mover based on pre-move characteristics including gender, career age, academic field (Scopus ASJC classification), number of publications, region of the source institution, and academic cohort.

The outcome variable of this analysis is the size of the individual professional network, measured as the number of unique first- and second-order co-authors accumulated prior to the mobility event (for movers) or at the matched career stage (for non-movers). As shown in Figure S2, movers and non-movers exhibit nearly identical distributions of network size, with 7.82 co-authors for non-movers and 7.80 for movers on average, and the difference is statistically insignificant. These results indicate that, conditional on observable characteristics, non-mobility cannot be attributed to systematically smaller professional networks. The findings mitigate concerns that our mobility-conditional analyses disproportionately capture highly network-endowed scholars. However, it is also important to note that this analysis does not rule out the role of unobserved characteristics (e.g., talent, preferences, family constraints), nor does it imply that networks do not causally affect the probability of moving.

8. Relative Prestige Analysis

Prior work on faculty hiring networks has shown that upward moves are far less common than downward or lateral moves, reflecting a strict prestige hierarchy (5). We test whether analogous hierarchical patterns are present in early-career scientific mobility by constructing a relative prestige measure defined as the difference between the log prestige of the target and source institution. The Leiden Ranking provides multiple sorting criteria, including collaboration, open access, and scientific impact. Within the scientific impact dimension, institutions can be sorted by different indicators. We use P(top 50%) as it captures scientific impact without conflating prestige with the collaboration patterns we aim to explain or with open access considerations. The resulting distribution (Fig. S3) is approximately symmetric and centred around zero. To classify movements while avoiding arbitrary ordinal rankings, we adopt a conservative, distribution-based threshold approach: moves below -0.15 standard deviations are classified as downward, moves above $+0.15$ standard deviations as upward, and the remainder as lateral. The ± 0.15 standard deviations threshold corresponds to a small standardized difference in institutional prestige, ensuring that only moves that are meaningfully distinct from zero are classified as upward or downward.

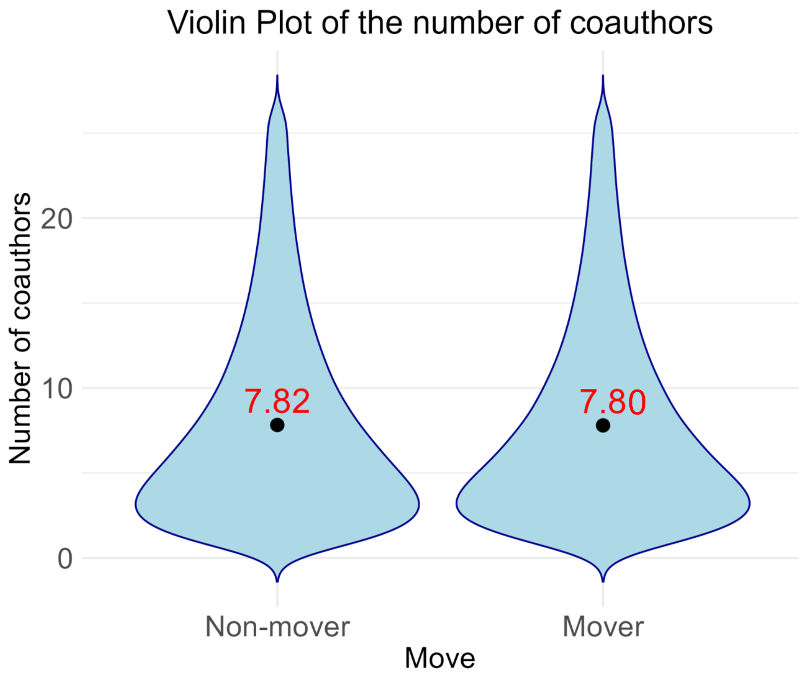

Fig. S2. Comparison of co-authorship network size between matched movers and non-movers. The figure shows the distribution of co-authorship network size. Red numbers indicate mean values for each group.

Using this definition, approximately 41.7% of first career moves are upward, 12.4% are lateral, and 45.8% are downward. These proportions differ markedly from faculty hiring networks, where upward moves are rare, reflecting the fact that early-career mobility is not governed by the same hierarchical constraints that characterize senior faculty hiring. Downward or lateral moves at this career stage often reflect labour market structure, postdoctoral placement dynamics, and field-specific opportunity distributions rather than long-term stratification.

Table S12 reports the distribution of individual-level and institutional-level connections across relative prestige classes. The prevalence of connection types varies only modestly across upward, lateral, and downward moves, and we do not observe the strong hierarchical asymmetries reported in faculty hiring networks.

Together, these results indicate that relative prestige, as measured using the Leiden Ranking does not function as the primary organizing principle for early-career scientific mobility in our data. We therefore retain absolute institutional prestige as our main predictor, as it is better aligned with both the career stage under study and the conceptual role of prestige in our analytical framework.

	Individual connection%	Institutional connection%	Both connection%	Neither connection%
Downward	18.9	16.5	23.4	41.2
Lateral	17.4	20.1	33.0	29.5
Upward	18.7	17.8	33.0	30.6

Table S12. Distribution of connection types exhibited by each relative prestige class.

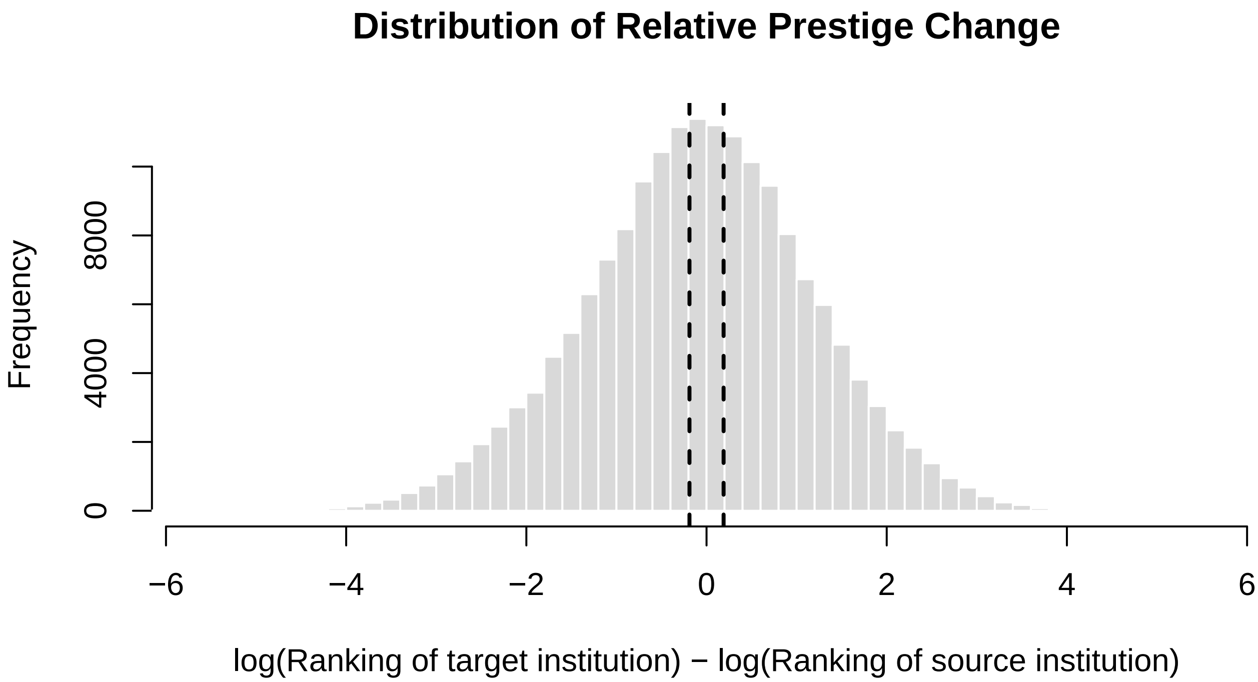

Fig. S3. Distribution of relative prestige differences between destination and source institutions, measured as log(Ranking of target institution) - log(Ranking of source institution). The distribution is approximately symmetric and centred around zero. Vertical dashed lines indicate the ± 0.15 standard deviation thresholds used to classify moves as downward (below -0.15σ), lateral (within $\pm 0.15\sigma$), and upward (above $+0.15\sigma$).

References

1. J Baas, M Schotten, A Plume, G Côté, R Karimi, Scopus as a curated, high-quality bibliometric data source for academic research in quantitative science studies. *Quant. Sci. Stud.* **1**, 377–386 (2020).
2. F Pedregosa, et al., Scikit-learn: Machine learning in Python. *J. Mach. Learn. Res.* **12**, 2825–2830 (2011).
3. JS Katz, BR Martin, What is research collaboration? *Res. Policy* **26**, 1–18 (1997).
4. G Laudel, What do we measure by co-authorships? *Res. Eval.* **11**, 3–15 (2002).
5. A Clauset, S Arbesman, DB Larremore, Systematic inequality and hierarchy in faculty hiring networks. *Sci. Adv.* **1** (2015).
